# A cross-sectional assessment of knowledge, attitude and practice among Hepatitis-B patients in Quetta, Pakistan

**DOI:** 10.1186/1471-2458-13-448

**Published:** 2013-05-06

**Authors:** Noman ul Haq, Mohamed Azmi Hassali, Asrul Akmal Shafie, Fahad Saleem, Maryam Farooqui, Abdul Haseeb, Hisham Aljadhey

**Affiliations:** 1 Department of Pharmacy, University of Baluchistan, Quetta, Pakistan/Discipline of Social and Administrative Pharmacy, School of Pharmaceutical Sciences, Universiti Sains Malaysia, Penang, Malaysia; 2 Discipline of Social and Administrative Pharmacy, School of Pharmaceutical Sciences, Universiti Sains Malaysia, Penang, Malaysia; 3 Department of Pharmacy, University of Baluchistan, Quetta, Pakistan/Discipline of Social and Administrative Pharmacy, School of Pharmaceutical Sciences, Universiti Sains Malaysia, Penang, Malaysia; 4 Faculty of Pharmacy, Universiti Teknologi MARA (UiTM), Penang, Malaysia/Discipline of Social & Administrative Pharmacy, Universiti Sains Malaysia (USM), Penang, Malaysia; 5 Faculty of Pharmacy, Umm Al Qura University, Makkah, 21955, Saudi Arabia; 6 College of Pharmacy, King Saud University, Riyadh, 11451, Saudi Arabia

**Keywords:** Hepatitis-B, Pakistan, KAP, Hepatitis-B patients

## Abstract

**Background:**

Hepatitis-B is a life threatening infection resulting in 0.6 million deaths annually. The prevalence of Hepatitis-B is rising in Pakistan and furthermore, there is paucity of information about Knowledge, Attitude and Practice among Hepatitis-B patients. Better disease related knowledge is important to have positive attitude and that will bring the good practices which will prevent the further spread of infection. This study aimed to evaluate knowledge, attitude and practice of Hepatitis-B Patients in Quetta city, Pakistan.

**Methods:**

A cross-sectional, descriptive study was undertaken with 390 Hepatitis-B patients attending two public hospitals in Quetta city, Pakistan. Knowledge, attitude and practice regarding Hepatitis-B were assessed using a pre-validated questionnaire containing 20, 7 and 8 questions for knowledge, attitude and practice, respectively. Descriptive statistics were used for elaborating patients’ demographic characteristics and mean scores for knowledge, attitude and practice of Hepatitis-B patients. Inferential statistics (Mann–Whitney *U* test and Kruskal Wallis tests, p < 0.05) were used to establish association between study variables. Spearman’s rho correlation was used to identify the association between the knowledge, attitude and practice scores.

**Results:**

Out of 390 patients, 223 (57.2%) were males, with the majority (136, 34.9%) in the age group of 38–47 years. Mean age of the study cohort was 32.6 ± 9.5 years. One hundred and four (26.7%) had primary level education, with 110 (28.2%) working in the private sector. The mean scores for knowledge, attitude and practice were 8.48 ± 2.7, 3.87 ± 1.2 and 2.37 ± 1.0, respectively. Education, locality and occupation were significantly associated with knowledge, attitude and practice scores. Significant positive linear correlations between knowledge-attitude (r = 0.466, p < 0.01) knowledge-practice (r = 0.221, p < 0.01) and attitude-practice (r = 0.224, p < 0.01) were also observed from the study results.

**Conclusion:**

The findings of this study indicate that Hepatitis-B patients lack a basic understanding of infection control and management. This can result in the further spread of Hepatitis-B infection. Extensive health education campaigns should be provided to the patients in the hospital as well as in community settings for rational control and management of the disease.

## Background

Hepatitis-B (HB) is a serious infectious disease of the liver, infecting 2 billion people around the globe; of these, 350 million will develop lifelong chronic infection [1]. An estimated 15–40% of chronic HB carriers are susceptible to develop liver cirrhosis and hepatocellular carcinoma [2,3]. HB is a life threatening infection resulting in 0.6 million deaths annually [4,5]. In Asia, 10 to 15 million individuals suffer from HB [6-8]. Similarly to what is reported worldwide, the prevalence of HB in Pakistan ranges between 7 to 20% based on different regions [6,9-14]. In urban areas of Pakistan, the prevalence of HB is reportedly lower (2-5%) than rural areas, where the prevalence is reported to range between 30-35% [11,13,14].

Knowledge, attitude, and practice (KAP) studies are representative of a specific population to collect information on what is known, believed and performed in relation to a particular topic. Knowledge, attitude, and practice are the most frequently used study tools in health-seeking behaviour research [15,16]. Knowledge is usually assessed to evaluate how patients’ knowledge corresponds to biomedical concepts [17]. Questions included in knowledge assessment are related to causes, symptoms, transmission and treatment and the management of the disease or infection condition under investigation [18]. Attitude is “a learned predisposition to think, feel and act in a particular way towards a given object or class of objects” [19]. Practices in KAP surveys usually inquire about the use of preventive measures or different healthcare options. In certain cases, it permits statements about actual practices as well as yielding information on people’s behaviours or on what they know should be done in a hypothetical scenario [20]. Therefore, measuring KAP is consequently critical to prevent the spread of infection.

Within this context, the health system in Pakistan consists of traditional public and private sectors. The public sector encompasses a wide network of dispensaries, basic health units, rural health centres and hospitals [21]. However, in the private sector, apart from some accredited outlets and hospitals, there is mushrooming growth of medical general practitioners and other complementary and alternative medicine (CAM) options (homoeopathic practitioners, traditional and spiritual healers, Greco-Arab healers, herbalists, bonesetters and hakims) [21]. Since CAM options provide easy and cheap treatment options with an easy access, the majority of patients, regardless of their disease or illness, seek CAM therapies as their primary choice of treatment. This leads to inappropriate or delayed healthcare, resulting in undesirable outcomes [22]. In addition, such practices also lead to the spread of infection among healthy societal members. At present, the prevalence of HB is rising in Pakistan and furthermore, there is paucity of information about KAP among HB patients. Therefore, the purpose of the present study was to assess KAP among HB patients in order to use the data in developing information, education and communication activities for the patients as well as community. Such activities will be helpful in the prevention and control of HB in a developing country like Pakistan.

## Methods

### Study design and settings

The study was designed as a questionnaire-based, cross-sectional analysis; the STROBE guideline was used to report the data [23]. Registered HB patients from two public hospitals (Sandamen Provisional Hospital and Bolan Medical Complex Hospital) in Quetta city, Pakistan were included for the study. Both of these hospitals are tertiary care institutes and, being public in nature, provide treatment to the majority of the population.

### Study sample, inclusion and exclusion criteria

Hepatitis-B is reported to affect 11% of the population of Quetta, Pakistan [10,13,14]. Therefore, a prevalence-based sample of 390 HB patients was selected (in total there were 429 registered HB patients in the area) [24,25]. The study was carried out from March 2011 to July 2011. Patients aged 18 years and above, with a confirmed diagnosis (HbsAG-positive) of HB, and familiar with Urdu (national language of Pakistan) were included in the study. Patients with co-morbidities, immigrants from other countries and pregnant women were excluded.

### Ethical approval

This study was performed according to the ethical standards for human experimentation [26]. The Joint Clinical Research Committee approved the study protocol (No: EA/NUH/1205-2009). Written consent was also taken from the patients prior to data collection. Patients were assured about the confidentiality of their responses and their right to withdraw from the survey at any time.

### Study instrument

A 35-item questionnaire comprised of four sections was used for data collection. In addition to the demographic data, 20 questions for knowledge, 7 questions for attitude and 8 questions for practices were designed to evaluate KAP among HB patients. Respondents were asked to answer in limited as well as multiple choice formats. The primary version of the questionnaire was developed through an extensive literature review in English [16,21,27]. The English version was later translated into Urdu by using standard translating procedures [28,29]. The Urdu version was tested for its reliability and validity. Internal consistency was assessed by using Cronbach’s alpha (α = 0.78) [30]. Face, content and convergent validity of the questionnaire was performed by experts at Discipline of Social and Administrative Pharmacy, School of Pharmaceutical Sciences, Universiti Sains Malaysia. The questionnaire was than piloted with 30 respondents for its acceptability and consistency. Few modifications were needed after the pilot testing. Data from the pilot study was not included in the final analysis. When the consistency and validity of the study questionnaire was stabilised, the instrument was made available for data collection.

### Statistical analysis

Descriptive statistics were used to illustrate respondents’ demographic characteristics. Categorical variables were measured as percentages while continuous variables were expressed as mean ± standard deviation. The Kolmogrov-Smirnov test was applied to determine the nature of response distribution. Inferential statistics (Mann–Whitney *U* test and Kruskal Wallis tests, p < 0.05) were used to assess the significance among study variables. Bonferroni post hoc adjustment was performed to detect the intergroup significance. Spearman’s rank correlation coefficient (p < 0.01) was used to evaluate the association between KAP. All analyses were performed using SPSS v. 16.

## Results

### Demographic characteristics

The demographic characteristics of the HB patients are presented Table 1. Mean age of the study cohort was 32.6 ± 9.59 years with males as the dominant gender (n = 223, 57.2%). One hundred and four (26.7%) had primary level of education and 160 (41.0%) were unemployed. Eighty three (21.3%) had a monthly income in between 10,001-15,000 Pakistani Rupees with 273 (70%) having urban residency. The major sources of information regarding HB were family/friend/neighbours (n = 144, 36.9%), followed by healthcare providers (n = 135, 34.6%).

**Table 1:** **Demographic characteristics of the study respondents** (**N** = **390**)

**Characteristics**	**N**	**%**
***Age*** (***32***.***61*** ± ***9***.***48***)		
18-27	58	21.8
28-37	125	32.1
38-47	136	34.9
48-57	35	9.0
57<	03	2.3
***Gender***		
Male	223	57.2
Female	167	42.8
***Education***		
Illiterate	19	4.9
Religious only	67	17.2
Primary	104	26.7
Metric	55	14.1
Intermediate	66	16.9
Graduation	55	14.1
Post-Graduation	24	6.2
***Occupation***		
Unemployed	160	41.0
Government Servant	34	8.7
Private Servant	110	28.2
Self Employed	86	22.1
Income*		
No Income	150	38.5
< Pk. Rs. 5000	50	12.8
5001-10000	35	9.0
10001-15000	83	21.3
>15001	72	18.5
***Locality***		
Urban	273	70.0
Rural	117	30.0
***Source of HB information***		
Newspapers and magazines	64	16.4
Health care providers	135	34.6
Family/friends/neighbours	144	36.9
TV, radio and internet	31	8.0
Religious leaders/teachers	03	0.8
HB information leaflets, brochures, posters etc.	13	3.3

*1 Pk Rs (Pakistani rupees) = 0.01172 USD (US dollars).

### Assessment of knowledge towards HB

Table 2 describes the current status of knowledge among HB patients. Knowledge was assessed by asking questions about aetiology, signs and symptoms, transmission, treatment and management of HB. Each response was scored as ‘yes’ or ‘no’. Knowledge score ranged from 20 (maximum) to 0 (minimum). A cut off level of < 11 was considered as poor whereas ≥ 11 was considered as adequate knowledge about HB. Knowledge scores for individuals were calculated and summed up to give the total knowledge score.

**Table 2:** Responses to Hepatitis B knowledge items

**Hepatitis B knowledge items**	**Yes N (%)**	**No N (%)**
Have you ever heard of a disease termed as Hepatitis?	383 (98.2)	7 (1.8)
Have you ever heard of a disease termed as Hepatitis B?	310 (79.5)	80 (20.6)
Is Hepatitis B a viral disease?	144 (36.9)	246 (63.1)
Can Hepatitis B affect liver function?	168 (43.1)	222 (56.9)
Can Hepatitis B cause liver Cancer?	105 (26.9)	285 (73.1)
Can Hepatitis B affect any age group?	103 (26.4)	287 (73.6)
The early symptoms of Hepatitis B are same like cold and flu (fever, running nose, cough)	98 (25.1)	292 (74.9)
Jaundice is one of the common symptoms of Hepatitis B?	148 (37.9)	242 (62.1)
Are nausea, vomiting and loss of appetite common symptom of Hepatitis B?	105 (26.9)	285 (73.1)
Are there no symptoms of the Hepatitis B in some of the patients?	95 (24.4)	295 (75.6)
Can Hepatitis B be transmitted by un-sterilized syringes, needles and surgical instruments?	152 (39.0)	238 (61.0)
Can Hepatitis B be transmitted by contaminated blood and blood products?	170 (43.6)	220 (56.4)
Can Hepatitis B be transmitted by using blades of the barber/ear and nose piercing?	139 (35.6)	251 (64.4)
Can Hepatitis B be transmitted by unsafe sex?	84 (21.5)	306 (78.5)
Can Hepatitis B be transmitted from mother to child?	99 (25.4)	291 (74.7)
Can Hepatitis B be transmitted by contaminated water/food prepared by person suffering with these infections?	146 (37.4)	244 (62.5)
Is Hepatitis B curable/treatable?	186 (47.7)	204 (52.3)
Can Hepatitis B be self-cured by body?	128 (32.8)	262 (67.2)
Is vaccination available for Hepatitis B?	240 (61.5)	150 (38.5)
Is specific diet required for the treatment of Hepatitis B?	291 (74.6)	99 (35.3)

Note: Knowledge was assessed by giving 1 to correct answer and 0 to the wrong answer. The scale measured knowledge from maximum 20 to minimum 0. Scores < 11 were taken as poor, ≥ 11 as adequate knowledge of Hepatitis B. Mean knowledge was 8.74 ± 2.7.

Out of the 390 HB patients, 298 (76.4%) were within the poor knowledge range whereas 92 (23.6%) showed adequate knowledge about HB. Poor knowledge was apparent in responses to questions relating to symptoms (questions 7–10) and the transmission of HB (questions 11–16). Correct response rates to these questions were 25.1%, 37.9%, 26.9% and 24.4% for symptoms and 39.0%, 43.6%, 35.6%, 21.5%, 25.4%, and 37.4% for the transmission of HB. The mean knowledge score for the entire study cohort was 8.48 ± 2.7.

### Assessment of attitude towards HB

Attitude towards HB was assessed by asking seven questions, as shown in Table 3. Each question was labelled with positive or negative attitude; the respondents were allowed to choose to only one response. A score of 1 was given to positive while 0 was given to negative attitudes with a score range of maximum of 7 to a minimum of 0. A cut off level of ≤ 4 was considered as negative whereas > 4 was considered as positive attitude towards HB.

**Table 3:** Responses to attitude toward Hepatitis B

**Hepatitis B attitude items**	**N**	**%**
***Have you ever thought you could get Hepatitis B?***		
Yes*	138	35.4
No	252	64.6
***What was your reaction when you found that you have Hepatitis B?***		
Fear*	138	35.4
Shame	104	26.7
Surprise	36	9.2
Sadness	112	28.7
***Who did you talk to about your illness?***		
Physician	67	17.1
Spouse	160	41.0
Parents	55	14.1
Child	24	6.2
Other relatives	10	2.6
Friends	70	17.9
No one^¥^	04	1.0
***What did you do when you knew that you have symptoms of Hepatitis B?***		
Went to health facility*	142	36.4
Went to a Hakeem	80	20.5
Went to a Homeopath	50	12.9
Went to a traditional healer	118	30.3
***When you had symptoms of Hepatitis B***, ***at what stage you came to the health facility?***		
Own treatment fails	184	47.2
After 3–4 weeks of the appearance of symptoms	95	24.2
Soon as I realize the symptoms are of Hepatitis B*	104	26.78
Will not go to physician	07	1.8
***How expensive do you think is the diagnosis and treatment of Hepatitis B?***		
Free	1	0.3
Reasonable	33	8.5
Somewhat expensive	137	35.1
Expensive	182	46.7
Don’t know^¥^	37	9.5
***What worries you most after you diagnosed with Hepatitis B***		
Fear of death	94	24.1
Fear of disease spread to family	53	13.6
Cost of treatment	71	18.2
Isolation from the society^¥^	172	44.1

^*^Positive attitude, ^¥^Negative attitude.

Note: Attitude was assessed by giving 1 to positive and 0 to negative attitude. The scale classified attitude as positive with score >4 and negative ≤4. Over all the respondents had a negative attitude towards Hepatitis B with mean score of 3.87 ± 1.2.

Out of the 390 HB patients, 309 (79.2%) were within the negative attitude range whereas 81 (20.8%) showed a positive attitude towards HB. The majority of the HB patients (n = 252, 64.6%) believed that they could never become infected with HB. One hundred and thirty eight (35.4%) HB patients stated that they felt fear when they found out that they were infected with HB. Two hundred and forty eight (73.6%) of the study respondents used CAM therapies for their infection before consulting a physician. However, respondents were ready to disclose their disease to their spouse (n = 160, 47.1%) and friends (n = 70, 17.9%). The mean score of attitude for the entire study cohort was 3.87 ± 1.2.

### Assessment of practices towards HB

Practices towards HB were assessed by asking six questions, as shown in Table 4. Each question was labelled with good or poor practice. A score of 1 was given to good while 0 was given to bad practice, with a score range of a maximum of 8 to a minimum of 0. A cut-off level of ≤ 5 was considered as negative whereas > 5 was considered as good practices towards HB.

**Table 4:** Responses to practices related to Hepatitis B

**Hepatitis B practice items**	**Yes N (%)**	**No N (%)**
Have you done screening for Hepatitis B?	07 (1.8)	383 (98.2)
Do you ask for screening of blood before transfusion?	94 (24.1)	296 (75.9)
Do you ask your barber to change blade/or for safe equipments for ear and nose piercing?	97 (24.9)	293 (75.1)
Do you practice safe sex?	156 (40.0)	234 (60.0)
Do you share food/utensils/water etc. with others	70 (17.9)	320 (82.1)
Do you avoid meeting people?	308 (79.0)	82 (21.0)
Will continue and complete the treatment against the infection?	354 (90.8)	36 (9.2)
Have you ever participated in health education program related to Hepatitis B?	24 (6.2)	366 (93.8)

Note: Practice was assessed by giving 1 to positive and 0 to negative attitude. The scale classified practice as good with score >5 and poor ≤5. Over all the respondents reported to have poor practice towards Hepatitis B with mean score of 2.37 ± 1.0.

Two hundred and sixty one (66.9%) patients were within the bad practice range whereas 129 (33.1%) showed good practice towards HB. The majority of the patients (n = 383, 98.2%) had never had HB screening before they became infected. Two hundred and ninety six (75.9%) patients had never inquired about blood screening or the safety of blood products before transfusion. Two hundred and ninety three (75.1%) respondents had never asked their barber to use a new blade, or for safe and clean equipment before nose and ear piercing. However, the majority of the patients (n = 308, 79.0%) revealed that they avoided meeting with people after becoming infected with HB. The mean score for HB related practices was 2.37 ± 1.0,revealing poor practices among study participants.

### Association of demographic characteristics and mean KAP scores

The association of demographic characteristics and mean KAP scores is presented in Table 5. Among the demographic variables, education level, occupation and area of residence (locality) were significantly associated with mean KAP scores (p < 0.05). Bonferroni adjustment using a general approach was used to investigate the significance among intergroup variables [31]. Furthermore, it was revealed that with regard to the educational variables, the illiterate group showed a significant relationship with the metric and bachelor level of education, the religious education group had a significant relationship with the bachelor level of education and the primary education group had a significant relationship with the intermediate and bachelor level of education. A significant difference was also found for occupation where unemployed respondents had a significant association with those having private or government jobs.

**Table 5:** **Mean scores of knowledge**, **attitude and practice**

**Description**	**N (780)**	**Knowledge score (Mean ± SD)**	**P value**	**Attitude score (Mean ± SD)**	**P value**	**Practice score (Mean ± SD)**	**P value**
Age (36.62 ± 9.597)*							
18-27	85	8.41 (2.8)	0.200	4.08 (1.2)	0.240	2.28 (0.9)	0.373
28-37	125	9.02 (2.8)		4.06 (1.2)		2.46 (1.0)	
38-47	136	8.46 (2.6)		3.67 (2.4)		2.43 (1.1)	
48-57	35	7.37 (2.5)		3.66 (1.1)		2.11 (0.9)	
>58	09	6.11 (2.1)		3.91 (1.2)		2.11 (0.9)	
Gender**							
Male	223	8.73 (2.6)	0.290	3.96 (1.2)	0.602	2.47 (1.0)	0.340
Female	167	8.14 (2.9)		3.79 (1.2)		2.24 (0.9)	
Education*							
Illiterate	19	6.26 (2.3)	***0***.***001***	3.16 (0.8)	***0***.***001***	1.79 (0.5)	***0***.***001***
Religious only	67	7.03 (2.5)		3.55 (0.9)		2.06 (0.9)	
Primary	104	7.81 (2.5)		3.77 (1.0)		2.00 (0.6)	
Metric	55	9.07 (2.5)		3.95 (1.2)		2.40 (0.8)	
Intermediate	66	9.10 (2.8)		4.18 (1.2)		2.39 (1.0)	
Graduation	55	9.74 (2.2)		4.29 (1.3)		3.05 (1.1)	
Post-graduation	24	11.37(1.8)		4.35 (1.2)		3.63 (1.2)	
Occupation*							
Unemployed	166	7.91 (2.8)	***0***.***001***	3.86 (1.2)	***0***.***001***	2.19 (0.9)	***0***.***001***
Government servant	34	10.41(2.2)		4.16 (1.4)		3.38 (1.3)	
Private servant	110	9.27 (2.7)		4.08 (1.1)		2.48 (1.0)	
Self employed	86	7.76 (2.3)		3.77 (1.1)		2.17 (0.8)	
Income*							
No income	150	7.87 (2.9)	0.131	3.87 (1.2)	0.269	2.20 (0.9)	0.145
< Pak Rs. 5000	50	8.92 (2.2)		4.02 (1.2)		2.22 (1.0)	
5001-10000	35	7.74 (3.3)		3.86 (1.0)		2.14 (0.7)	
10001-15000	83	9.18 (2.4)		3.94 (1.2)		2.41 (1.0)	
>15001	72	8.97 (2.6)		3.95 (1.2)		2.90 (1.2)	
Locality**							
Urban	273	9.07 (2.6)	***0***.***001***	3.98 (1.2)	***0***.***001***	2.50 (1.0)	***0***.***001***
Rural	117	7.10 (2.6)		3.83 (1.1)		2.07 (0.9)	
**Total**	**390**	**8**.**48** (**2**.**7**)		**3**.**87** (**1**.**2**)		**2**.**37** (**1**.**0**)	

* Kruskal Wallis Test (p< 0.05).

** Mann Whitney Test (p< 0.05).

### Correlation between KAP

Spearman rank correlation revealed significant positive linear correlations between knowledge-attitude (r = 0.446, p < 0.01), knowledge-practice (r = 0.221, p < 0.01) and attitude-practice (r = 0.224, p < 0.01). This result reaffirms the relationship between knowledge attitude and practice of infection control measures, as shown in Table 6.

**Table 6:** **Correlation between knowledge**, **attitude**, **and practice scores**

**Variable**	**Correlation coefficient**	P-value*
Knowledge-Attitude	0.446	<0.01
Knowledge-Practice	0.221	<0.01
Attitude-Practice	0.224	<0.01

*Correlation significant at 0.01 levels (2 tailed).

## Discussion

The results of the current study reveal that HB patients have poor KAP towards their disease. A small percentage of patients actually know about the transmission of HB; this lack of knowledge about HB transmission can be attributed to a rise in the frequency of HB. This finding is in line with that which was reported earlier, where poor knowledge levels of different populations from different regions were reported [21,32-37]. However, such studies were concerned with the knowledge of the general or healthy population and there are no data on the knowledge of HB patients.

The results of the present study revealed that, although all the patients had access to healthcare professionals, the primary source of information regarding HB was through family, friends and neighbours. Therefore, we can conclude that poor KAP scores are correlated with advice taken from an unreliable source. A primary reason of this attitude is linked to the lack of communication between the healthcare professional and the patient, meaning that the patient may not receive adequate information. Healthcare professionals should disseminate information regarding HB and present themselves as a primary source of information for HB patients. This will provide evidence-based knowledge to HB patients, which will improve HB patients’ compliance towards their treatment and prevent the further spread of infection.

The mean attitude score was found to be in the negative range. Before being diagnosed with HB, the majority of patients believed that they could never get the infection. Perceived susceptibility or a viewpoint of how vulnerable a person considers himself/herself to getting a disease can influence one’s attitude in taking certain actions [38]. In addition, the majority of patients revealed the use of complementary and alternative medicines after they became infected with HB before consulting a physician. Homoeopaths and traditional/spiritual healers were the treatment of choice until there was no improvement in the signs and symptoms of HB. Consulting physicians was sought as the last resort, when all other healing systems had failed to provide a cure. This is in line with that which was reported earlier in Pakistan [39]. The delay in seeking medical treatment results in the further deterioration of the condition and can cause the spread of infections to other healthy individuals as well. Nearly half of the patients perceived HB treatment as expensive, which could be one reason for using alternative treatments. Within this context, poverty, cultural beliefs and perceived severity of illness could be additional reasons for seeking alternative methods of treatment. In developing countries like Pakistan, access to traditional healers is more economical than seeking treatment at medical healthcare facilities [39].

Isolation from society was the biggest fear after being diagnosed with HB in patients in the current study. Isolation describes the absence of social interactions in society and results in several behavioural changes. The phenomenon of isolation has been widely studied among patients with mental health problems [38]; however, this phenomenon is not well explored among HB patients from conservative societies like Pakistan. Such behavioural changes may also affect patients’ health-related quality of life which is evident from literature [40]. It is interesting to note that the majority of patients also reported that they avoid meeting people; this is bad practice as it could lead them to become self-isolated. Therefore, their fear that they will be isolated could be because they themselves avoid meeting with people rather than society making them isolated.

Patients in the current study also showed poor practice towards HB. Only a small number of patients appeared for HB screening before they were diagnosed with infection. The majority of the patients were not concerned about the safety measures which exposed them to the danger of spreading HB infection within their social circle. Poor practices were also evident from practicing unsafe sex and not taking safety measures while seeking services at barber shops. These poor practices impose a danger to other healthy individuals in society. Thus, all HB diagnosed patients should be informed regarding the importance of safe practice and the dangers of spreading the infection to healthy individuals. Only a very small number of the patients had attended a health education program, and they reported a lack of information about educational campaigns in their areas. Educational programs are strongly recommended for healthy individuals, as well as for HB patients. Mass media should be recognised and introduced as an effective educational tool for HB patients with an aim of a better understating of HB.

In the current study, the education level, occupation and area of residence (locality) were the significant demographic factors associated with the mean KAP scores. Although education level was reported to be significantly associated with KAP scores in other studies from different parts of the world [34,41], the literature do not report a relationship between occupation or residence and KAP of HB patients.

The positive correlations between knowledge-attitude, knowledge-practice and attitude-practice in this study reaffirm the relationship between knowledge attitude and practice with infection control measures. It is concluded that adequate knowledge can lead to a positive attitude, resulting in good practices. The findings are in line with the results of some previous studies; however, such results were a representation of a healthy population and no data are reported from the perspective of HB patients [21,42].

The reported positive correlations are explainable by the theory of Reasoned Action. A person’s intention to a specific behaviour is a function of their attitude towards that behaviour. Furthermore, the attitude toward the behaviour is determined by the person’s belief that a given outcome will occur if he/she will perform the behaviour [43]. Within this context, in the current study, the practices related to HB as a performed behaviour were dependent on the attitude of the patients towards their disease condition. In addition, the attitude was shaped because of the knowledge that the patient possesses regarding HB infection. Hence, it is concluded that correct knowledge brings about a positive attitude and this positive attitude brings about a positive change in the practices of patients.

## Conclusion

The findings of this study indicate that HB patients lacked an understanding of infection control and management. Extensive health education campaigns should be provided to HB patients in both hospital and community settings using a patient-centric approach. Physicians, pharmacists and nurses should play a role in developing a collaborative care model to provide education to the patients. Empowering the patients will be helpful in disease management as well as in controlling the further spread of infection to the healthy population.

### Limitations

The study is as a cross sectional study on outpatients in public hospitals that are usually approached by low to middle income population. Whereas, the high income group usually uses these facilities in emergency only. Hence the results of our research may not represent the entire population.

## Competing interests

The authors have declared no conflict of interest. No funding was received for this study.

## Authors’ contributions

NH and FS conducted the survey and drafted the initial manuscript. MAH and AAS designed and supervised the study. MF, AH and HA helped in statistical analysis, interpretation and manuscript revision. All authors read and approved the final manuscript.

## Pre-publication history

The pre-publication history for this paper can be accessed here:

http://www.biomedcentral.com/1471-2458/13/448/prepub
